# Predicting VTE in Cancer Patients: Candidate Biomarkers and Risk Assessment Models

**DOI:** 10.3390/cancers11010095

**Published:** 2019-01-15

**Authors:** Silvia Riondino, Patrizia Ferroni, Fabio Massimo Zanzotto, Mario Roselli, Fiorella Guadagni

**Affiliations:** 1 Interinstitutional Multidisciplinary Biobank, IRCCS San Raffaele Pisana, 00166 Rome, Italy; silvia.riondino@sanraffaele.it (S.R.); fiorella.guadagni@sanraffaele.it (F.G.); 2 Department of Systems Medicine, Medical Oncology, University of Rome Tor Vergata, 00133 Rome, Italy; mario.roselli@uniroma2.it; 3 Department of Human Sciences & Quality of Life Promotion, San Raffaele Roma Open University, 00166 Rome, Italy; 4 Department of Enterprise Engineering, University of Rome “Tor Vergata”, 00133 Rome, Italy; fabio.massimo.zanzotto@uniroma2.it

**Keywords:** venous thromboembolism, biomarkers, clinical decision systems, risk assessment models, machine learning

## Abstract

Risk prediction of chemotherapy-associated venous thromboembolism (VTE) is a compelling challenge in contemporary oncology, as VTE may result in treatment delays, impaired quality of life, and increased mortality. Current guidelines do not recommend thromboprophylaxis for primary prevention, but assessment of the patient’s individual risk of VTE prior to chemotherapy is generally advocated. In recent years, efforts have been devoted to building accurate predictive tools for VTE risk assessment in cancer patients. This review focuses on candidate biomarkers and prediction models currently under investigation, considering their advantages and disadvantages, and discussing their diagnostic performance and potential pitfalls.

## 1. Introduction

Venous thromboembolism (VTE) represents a multifactorial disease that encompasses two main clinical entities, Deep Vein Thrombosis (DVT) and Pulmonary Embolism (PE).

The annual incidence rate of VTE varies greatly among ethnicity, ranging from 104 to 183 per 100,000 person-years in Europeans, and being higher in African Americans, and lower in Asians [1]. The rates of both DVT and PE increase with age [1] and, depending on the presence or not of a well defined clinical condition, it may occur either as a ‘provoked’, or as an “unprovoked’ phenomenon. Acquired (environmental) and genetic risk factors often coexist, thus contributing to enhance VTE risk [2]. The recognized acquired conditions that contribute to VTE development include surgery, hospitalization for acute illness, trauma or fracture, immobility, cancer (either occult or active) and its treatment, infections, obesity, increased patient’s age, pregnancy and post-partum period, oral contraception, and hormone therapy.

In hospitalized patients, either surgical or non-surgical, VTE represents one of the major causes of morbidity, disability, and mortality, and often requires an adequate thromboprophylaxis that may extend beyond the post-hospital discharge period [3]. However, VTE prevention represents a double-edged sword for clinicians that are faced with the possible consequences of an excess inhibition of coagulation that leads to hemorrhages. Indeed, if on one hand the appropriate use of antithrombotic agents is aimed at reducing both the occurrence and recurrence of VTE, on the other hand, the bleeding risk might be elevated, especially in the elderly and in acutely ill medical patients, who present with multiple co-morbidities and polytherapy that can interact with anticoagulant drugs [4]. All these considerations are particularly pertinent for cancer patients, who are in a condition of hemostatic unbalance toward a hypercoagulability state due to a sub-clinical activation of blood coagulation [5].

VTE arises as a frequent complication of cancer, occurring in up to 20% of cancer patients [6], is often incidentally diagnosed at time of restaging [7] in a percentage of cases that can reach approximately 50% [8] and it is associated with a rate of mortality of about 20–30% [9,10]. Moreover, the majority of cancer patients are under active anti-neoplastic treatment, and this represents an adjunctive risk factor for VTE development, due to both a direct damaging effect on endothelial cells and the amplification of the prothrombotic potential of cancer cells [11,12]. Of note, cancer-associated VTE represents an important medical challenge, since oncology patients concomitantly display higher rate of both bleeding, especially female [13] and VTE recurrence [14] and this highlights the need for a careful evaluation of the risk/benefit assessment of anticoagulant prophylaxis.

Thus, in outpatient chemotherapy candidates, the question of the benefit of primary thromboprophylaxis arises as an important issue. In this regard, current guidelines recommend antithrombotic prophylaxis for patients with cancer who are hospitalized for acute medical illness, while leaving aside all the group of out-patients receiving anti-cancer therapies, for whom routine prophylaxis is not recommended, except in selected categories of patients with solid tumors at high risk of thrombosis after careful assessment and discussion with the patients themselves [15,16,17,18,19,20,21]. This underlines the importance of managing accurate clinical predictive tools, built on clinical variables and/or associated biomarkers, to predict the risk of cancer related VTE.

In the past ten years, several clinical decision models have been designed to guide physicians in VTE risk assessment in the oncologic setting. Among them, the first and most widely used is the Khorana score [22], which aims at identifying ambulatory cancer patients at increased risk of VTE during chemotherapy. This model takes into account the site of cancer (distinguished in very-high risk, high-risk and other, lower-risk, cancer sites), thrombocytosis, leukocytosis, low hemoglobin levels, and a body mass index (BMI) ≥35 kg/m^2^ [22]. On the basis of the obtained score, patients are attributed to three categories with low-, intermediate- or high-risk for VTE during chemotherapy. However, even though the Khorana score is a user-friendly predictor, it is strongly dependent on tumor type and does not consider treatment-related factors influencing VTE development. Consequently, its external validation was not univocal [11,23,24], the major weakness being represented by a high proportion of patients (>50%) falling into the intermediate risk category that makes its clinical applicability suboptimal [11,23]. Other scores have, thus, attempted to increase the Khorana score predictive performance introducing different variables, such as soluble biomarkers (as in the Vienna Cancer and Thrombosis Study-CATS-score) [25], clinical features (as in the CONKO score, specifically developed for lung cancer) [26] or the use of specific chemotherapy regimens (as in the PROTECHT score) [27].

A big step forward in the decision making process, came from Artificial Intelligence (AI). Indeed, in recent years, the approach to prediction has substantially changed: global approaches have been pushed by a growing availability of electronic health records (EHRs) and by the consequent demand to provide precision medicine. In this context, AI would represent a sound instrument to build risk assessment tools, or decision support systems, in general. This review focuses on candidate biomarkers and prediction models for VTE risk assessment currently under investigation, considering their advantages and disadvantages, and discussing their diagnostic performance and potential pitfalls. The feasibility of an AI approach for VTE risk prediction in chemotherapy-treated cancer patients is further described in the appropriate section.

## 2. VTE Biomarkers

Many candidate biomarkers have been proposed to build strong clinical- as well as AI-based decision models for VTE risk assessment. Most of them relate to the pro-coagulant status that is associated with cancer and are represented by coagulation activation products. Beside these, various inflammatory or biochemical parameters have also been proposed, generally based on their significance as risk factors in arterial thrombosis. This section reviews the most important candidate biomarkers so far identified, most of which have been used to define/implement the currently available models.

**Figure 1:**
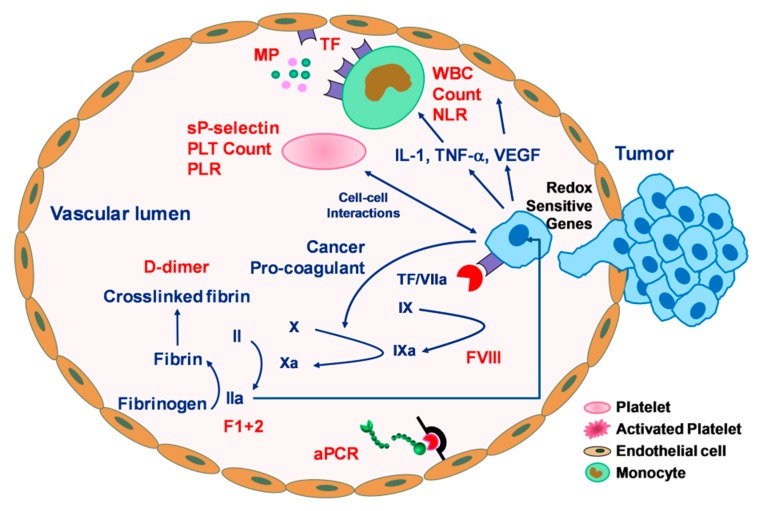
Graphical summary of the mechanism of tumor-induced coagulation cascade and relevant biomarkers at various stages of the pro-coagulant processes. Tumor cells express tissue factor (TF) and “cancer pro-coagulant” (CP). TF binds to activated factor VII (VIIa), forming a complex (TF/VIIa) that initiates coagulation cascade by activating factor IX and X, with consequent thrombin generation and fibrin formation. CP directly activates factor X. Thrombin, in turn, triggers platelet (PLT) activation with subsequent release of platelet granule content and amplification of the whole activatory process. Tumor cells may also interact with vascular cells (monocytes, platelets, endothelial cells) either directly (through membrane interactions) or indirectly (through cytokine release, prompted by activation of redox sensitive genes). Activated vascular cells release microparticles (MPs) with pro-coagulant activity in the circulation. Candidate biomarkers for VTE prediction are highlighted in red. aPCR: resistance to activated Protein C; F1+2: Prothrombin fragment; IL-1: interleukin-1; NLR: neutrophil/lymphocyte ratio; PLR: platelet/lymphocyte ratio; TNF-α: tumor necrosis factor-alpha; VEGF: vascular endothelial growth factor; WBC: white blood cells.

The epidemiology of VTE in cancer can be ascribable to different, interrelated, factors being dependent on the patient him/herself, the cancer (site and stage) [28,29], and the treatment [29,30,31], including radiotherapy [32], that sum up the additional risks typical of VTE such as immobilization, infection, and surgery. All cancer-associated factors contribute to enhance the pro-thrombotic state arising from alterations in the haemostatic system, stasis and blood flow slowdown, endothelial dysfunction and vascular inflammation, typical of a neoplastic condition in which the coagulation process and the inflammatory system are simultaneously stimulated [33]. Indeed, tumor cells can activate the coagulation cascade either through the production of pro-coagulant molecules, such as tissue factor (TF) [34,35] and “cancer pro-coagulant” (CP) [36], or by inducing a pro-coagulant phenotype in blood cells with which they interact, such as monocytes, platelets, and endothelial cells (Figure 1) through the expression of various adhesion molecules, including two selectin ligands to platelets (P-selectin) or endothelial cells (E-selectin) [37], both concurring to facilitate tumor cell invasion and metastasis [38,39,40]. The production of these molecules leads to thrombin generation, fibrin formation [41], and platelet activation [42].

**Figure 2:**
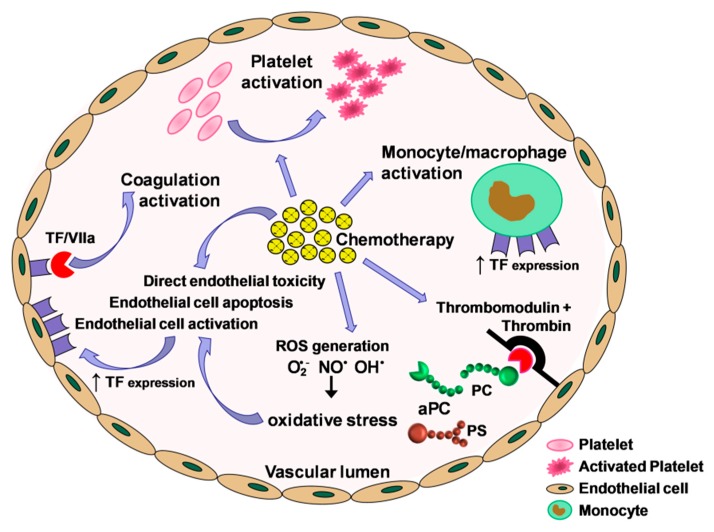
Effects of chemotherapy on coagulation activation. Chemotherapy may cause incongruous activation of hemostasis through various mechanisms: direct endothelial cell toxicity and apoptosis; vascular cell activation, resulting in tissue factor (TF) exposure; production of reactive oxygen species (ROS); unbalance of factors involved in the control of the coagulation cascade, with an impairment of the protein C (PC)/protein S (PS) anticoagulant pathway; activation of the monocyte/macrophage system and platelets. aPC: activated Protein C; (O_2_^•−^): superoxide anion; (OH^•^): radical hydroxyl radical; (NO^•^): nitric oxide; TF: tissue factor.

Treatment-related factors also play an important role, as demonstrated by the observation that VTE risk varies greatly among patients and even within the same patient over the course of the disease and in association with the different therapeutic interventions (from cancer surgery to adjuvant treatment and, eventually, metastatic treatment). Chemotherapy is among the causes frequently associated with a significantly increased risk of VTE especially in the first 3–6 months of therapy [29,43,44], owing to an inappropriate activation of hemostasis, either due to induction of procoagulant activity [45] or to downregulation of the anticoagulant protein C/protein S (PC/PS) pathway [44] (Figure 2). The occurrence of an acquired activated protein C (APC) resistance predictive of VTE during chemotherapy has been demonstrated evaluating the early changes of APC function [46]. In particular, platinum-based regimens have been found to be significantly associated with an acquired thrombophilic condition [31].

Accordingly, many predictive biomarkers have been proposed to improve VTE risk prediction to correctly identify the right subgroup of patient candidates for thromboprophylaxis (Figure 1), avoiding unnecessary use of anticoagulation that may lead to bleeding complications.

Although D-dimer remains the most common clinically used biomarker, other assays of hypercoagulability have been considered in risk assessment. However, no specific biomarker has been validated as a predictor for cancer-associated VTE so far, due to a poor feasibility in routine clinical practice, lack of standardization, lack of homogeneous and appropriate reference values, requirement of skilled personnel and of advanced techniques laboratories, or because their analytical measurement has not yet been introduced in the routine laboratory evaluation. Presently, D-dimer is the only factor whose determination has been introduced into routine practice and that might be employed as a possible VTE risk predictor.

### 2.1. D-Dimer

D-dimer has the most robust background as a marker of an ongoing fibrinolytic process. D-dimers are fragments produced when plasmin cleaves fibrin, thus representing the expression of fibrin formation and degradation occurring during the fibrinolytic activity of clot breakdown. Available tests for D-dimer quantification include enzyme-linked immunosorbent assays (ELISA), enzyme-linked immunofluorescence assay (ELFA), latex-enhanced immunoturbidimetric, and whole-blood point of care [47]. ELISA methods have a high sensitivity for low levels of D-dimer, and are thus considered the reference test. However, both ELISA and ELFA methods are time consuming, require specialized personnel, and cannot be used as bed-side testing, although ELFA can be performed on single samples. The introduction of point of care (POC) devices for D-dimer measurement has undoubtedly improved the turnaround time of D-dimer testing as compared to ELISA and several POC devices for determination of D-dimer are commercially available [48,49]. However, despite its high sensitivity, D-dimer testing might prove less specific, since, its levels increase in many conditions, such as, infection, surgery, pregnancy, and cardiovascular disorders that lower D-dimer specificity as a marker of an ongoing thrombosis and limit its efficacy to rule-in a thromboembolic episode [50,51]. Conversely, D-dimer has a high negative-predictive value, allowing the exclusion of an ongoing process of clot formation. Notably, D-dimer levels increase with age [52,53], and it has been suggested that an age-adjusted cut-off value should be used in patients 50 years, or older, by multiplying the patient’s age by 10 μg/mL [54,55], a hypothesis that is currently under investigation (ClinicalTrials.gov; NCT02384135).

Several studies considered D-dimer in the prediction of VTE risk in cancer patients [56,57,58,59] and its determination has been also proposed in those scheduled to receive active cancer treatment, since pretreatment levels of this biomarker were correlated with chemotherapy-associated VTE [60]. The value of D-dimer determination, prior to cisplatin-based chemotherapy start, in predicting VTE occurrence has proven effective in lung cancer outpatients, in which D-dimer levels above a locally established cutoff had a positive-predictive value of 31% [61]. Moreover, pretreatment D-dimer levels proved to be an independent risk factor of VTE in chemo-naïve patients with primary pancreatic adenocarcinoma [62] or in advanced gastric cancer patients receiving palliative chemotherapy with regimens that included fluoropyrimidine plus platinum, taxanes or irinotecan [63,64]. A recent meta-analysis performed to investigate the relationship between elevated D-dimer levels and VTE risk of ovarian cancer demonstrated that high D-dimer levels could predict both disease progression and VTE risk in this setting of patients [65].

Of interest, plasma D-dimer levels showed an association with disease state, prognosis, and the risk of VTE, not only at diagnosis, but also during the course of antineoplastic treatment [66].

### 2.2. Soluble P-Selectin

Soluble P-selectin (sP-sel) is emerging as a novel biomarker for the diagnosis of VTE [67] due to its association with vascular and thrombotic diseases [68]. sP-sel derives from the adhesion molecule P-selectin, contained in the α-granules of platelets and the Weibel–Palade bodies of endothelial cells [69]. Following platelet activation, P-selectin is expressed on the surface membrane and then shed by cleavage [70]. Despite its presence in endothelial cells, platelets are currently considered as the major source of circulating sP-sel in healthy individuals [71], suggesting its role as a reliable marker of in vivo platelet activation [72,73].

sP-sel determination is generally performed by ELISA [74], with all the limitations reported above. Furthermore, sP-sel normalization by platelet count should be considered—in order to reduce potential biases due to low platelet counts in thrombocytopenic patients—although sP-sel levels and platelet count did not show any relevant correlation in the study by Ay et al. [75]. The latter was the first clinical evidence that high plasma sP-sel levels may represent an independent VTE risk predictor in cancer patients [75]. In details, patients with sP-sel levels higher than the 75th percentile had a risk of VTE 2.6 times higher than those with lower levels (95% CI, 1.4–4.8), and a probability of developing VTE 6 months after study inclusion of 11.9% vs. 3.7% [75]. The same Authors later reported that patients who develop VTE have elevated sP-sel, D-dimer, Factor VIII (FVIII), and F 1+2 levels over the entire follow up period [76].

### 2.3. Microparticles

Microparticles (MPs) are membrane vesicles derived from apoptotic or activated cells, formed by outward extrusion of the plasma membrane and subsequently released following cytoskeletal proteolytic cleavage [77]. MPs contain several surface proteins including TF [78] and phosphatidylserine (PS) that account for their procoagulant activity [79]. Monocyte-derived MPs trigger coagulation predominantly via TF [80], while platelet-derived MPs promote thrombus propagation both by exposing PS on their surface and by initiating thrombin generation independently of TF and the extrinsic pathway, in an FXII-dependent manner [80,81].

Among the methods, flow cytometry, electron microscopy, atomic force microscopy, dynamic light scattering, measurement of TF antigen levels, and functional assays, are equally employed, with different advantages/disadvantages. For instance, platelet-derived MPs are commonly detected by flow cytometry [82], using a technique that allows the cellular origin of the MPs to be established but provides no information on their activity. Conversely, functional assays exploring the procoagulant activity of MPs have a high sensitivity, but do not allow MPs’ cellular origin to be defined [78]. Functional assays could be either PS-dependent or TF-dependent, and an association between tumor-derived TF^+^-MPs and VTE has been found in cancer patients. Indeed, cancer cells themselves may release procoagulant MPs and circulating tumor cell-derived TF^+^-MPs may trigger venous thrombosis formation in the absence of vessel injury through the formation of coagulation complexes [78,83].

However, despite the observation of increased MPs in patients with cancer-associated thrombosis [84,85,86,87,88,89], compared to those without [90], and although many studies linked elevated levels of MPs with future occurrence of thrombosis [91,92], others failed to demonstrate their role as predictive biomarkers [86,93]. These discrepancies might be partially explained by the poor standardization of analytical methods for MPs detection [94]. Indeed, although the model of MPs is fascinating, clinical research on MPs is biased by the variations in pre-analytical conditions of the currently available detection methods, that makes the results of plasma MP measurements widely variable [95,96].

### 2.4. Inflammatory Markers

Elevated levels of several interleukins (ILs) have been reported in cancer (resulting either from direct tumor production or from the underlying inflammatory process), depending on both type and stage of the disease [97]. IL-6 and IL-8, both capable of stimulating angiogenesis [98], have been associated with unfavorable outcome in patients with various cancer types, and with VTE occurrence [99]. A mild correlation between IL-6 or IL-1β levels and VTE was observed in pancreatic cancer patients [99], while a stronger association was reported for IL-6 and ovarian cancer [100]. In this setting, IL-6 has proven effective in favoring immune system escape by providing a platelet protective shield to tumor cells [101], stimulating platelet production [102], providing growth factors, and promoting tumor angiogenesis via vascular endothelial growth factor (VEGF) [100], all concurring to induce a prothrombotic condition.

Tumor-derived cytokines are also capable of interfering with the hemostatic balance, in particular with the anticoagulant pathway [103]. Indeed, acquired (inflammatory) APC resistance is common among patients with solid tumors, representing a more important risk factor for VTE in cancer than in non-malignant conditions [104,105]. Moreover, a decline in functional PC activity was demonstrated at mid-therapy [43], leading to an acquired APC resistance [106,107] which was predictive of VTE [108] and completely reversed at the end of chemotherapy [106,109]. In particular, tumor necrosis factor-α (TNF-α) has been shown to cause a dysfunction in the APC system, resulting in acquired APC resistance and increased VTE risk in metastatic colorectal cancer (mCRC) treated with chemotherapy [110]. Of interest, TNF-α has been proposed as a risk determinant for VTE in a sub-study of the Leiden Thrombophilia Study, based on the demonstration that individuals with detectable plasma TNF-α levels had a 2-fold increased VTE risk [111].

On the other hand, the acute phase reaction that accompanies cancer, may also cause the elevation of other pro-coagulant proteins, in particular the coagulation factor VIII (FVIII) [112,113]. Activated FVIII (FVIIIa) serves as a cofactor for the activation of the common pathway of the coagulation cascade and the conversion of prothrombin to thrombin. Initial evidence of an association between high levels of FVIII and risk of VTE came from the Leiden Thrombophilia Study and VTE [113], but were soon confirmed by others [114,115,116,117,118]. As in the case of D-dimer, elevated Factor VIII levels showed an association with the risk of VTE as well as patient’s prognosis, not only at diagnosis, but also during the course of antineoplastic treatment [66].

### 2.5. Routine Laboratory Parameters

Several parameters, associated with inflammation, whose analysis is routinely performed in laboratory practice, have been suggested to represent surrogate predictive markers of cancer-associated risk of thrombosis, and some of them have been included in risk assessment models (RAMs). Among the easiest to be collected stand blood cells counts, whose elevation represents a non-specific response to cancer-related inflammation.

#### 2.5.1. Hematological Parameters

Platelets—Platelets have long been demonstrated to play a key role not only in the process of metastasis dissemination but from the very beginning of tumor growth. Platelets store numerous inflammatory mediators in their granules and release them upon activation thus contributing to malignancy progression, angiogenesis, and tumor cell dissemination [11,119,120]. The released inflammatory mediators trigger leukocyte and endothelial cell activation, with subsequent aggregate formation [121]. Novel interesting observations, assign a role to cancer cells in modifying both physiology and phenotype of platelets and platelet RNA profile, thus contributing to the pro-thrombotic manifestations in cancer patients [122,123].

The evidence of independent associations between elevated platelet count and occurrence of VTE in patients with newly diagnosed cancer [25,124,125,126,127,128,129] or under active anti-cancer treatment [125] has provided support to investigate this parameter for VTE risk prediction [22]. The role of platelets in cancer-induced VTE is further supported by the independent findings by our research group [130] and by Riedl et al. [131] showing that mean platelet volume (MPV)—a marker of platelet activation elevated in arterial thrombosis—is significantly associated with VTE development, declining during the first three months of chemotherapy and reverting to baseline at the end of treatment, possibly as a result of drug-induced platelet activation and destruction [130]. Of interest, the predictive value of MPV was lately confirmed in patients with non-Hodgkin [132] or large B-cell lymphoma [133], especially when incorporated into VTE-RAMs [132].

Leukocytes—Similar to platelets, leukocytes also represent a link between cancer, thrombosis, and inflammation and their complete or differential count has, thus, been proposed as a predictive marker for cancer-associated VTE [22,25,134]. Pivotal studies demonstrated that an elevated white blood cell (WBC) count was associated with a significantly higher risk of VTE—or its recurrence—in cancer patients, suggesting that leukocytes may play a causal role in cancer-associated VTE rather than only representing an epiphenomenon of cancer-associated low-grade inflammation [22,135,136]. Other studies demonstrated the VTE risk predictive value of absolute neutrophil [137], or monocyte counts [137,138], but not of lymphocyte counts [137].

As stated above, the pathophysiological significance of these associations may be found in the entangled relationship occurring between cancer growth and progression, low-grade inflammation and thrombosis: activated monocytes can release TF^+^MPs, while activated neutrophils can release DNA, generating highly thrombogenic neutrophil extracellular traps (NETs). Platelet-derived MPs may further contribute to the procoagulant potential [139]. Nonetheless, their clinical significance in cancer is far from being fully elucidated.

Blood cell ratios—Recently, based on the notion that high neutrophil and platelet counts reflect inflammation, while low lymphocyte counts may be considered as a sign of poor general health and physiologic stress, platelet/lymphocyte ratio (PLR) and neutrophil/lymphocyte ratio (NLR) have been proposed as potentially useful prognostic parameters in cancer patients, as they integrate the detrimental effects of thrombocytosis or neutrophilia and lymphopenia [140].

PLR and NLR can be easily calculated from the differential blood count and have long been associated with adverse clinical outcome in cancer patients. Moreover, NLR and, to a higher extent PLR, have been found to be significantly elevated prior to chemotherapy in those patients who later developed VTE [134,141,142], and proved capable of identifying high-risk patients falling within the intermediate VTE class of risk according to Khorana [134]. These results, however, were not confirmed in a subsequent study, showing that both elevated PLR and NLR were independently associated with a twofold increased risk of mortality, but not VTE [143]. No data are currently available for monocyte/lymphocyte ratio.

#### 2.5.2. Biochemical Parameters

Other routinely analyzed biochemical parameters have been sporadically associated with the risk of VTE occurrence. For example, serum albumin—a negative acute phase reactant—has been proposed as a marker for VTE risk [144,145], which increased continuously with decreasing levels of albumin [144].

Other studies pointed out the possible predictive value of metabolic parameters, such as glycemic indexes or blood lipids. In particular, we recently demonstrated that evaluating glucose metabolic asset prior to chemotherapy may allow for VTE risk stratification in breast [146] or gastrointestinal cancer [147], independently of type 2 diabetes, overweight/obesity, or other well known risk factors. The possibility of a causal link between impaired glucose metabolism and VTE occurrence is biologically plausible and supported by the experimental finding that, in healthy non-diabetic subjects, increased blood glucose levels enhance blood coagulation [148].

On the other hand, based on the current knowledge that high-density lipoproteins (HDL) exert a protective effect by inhibiting vascular inflammation and enhancing endothelial function, we recently hypothesized that reduced HDL-cholesterol (HDL-C) levels might represent a risk factor for VTE onset in cancer outpatients receiving chemotherapy [149]. Indeed, patients with low HDL-C levels prior to chemotherapy start had a three-fold higher risk of developing VTE, independently of BMI [149]. The association between HDL-C and VTE risk in cancer patients was indirectly confirmed by subsequent studies demonstrating that patients under statins had a lower risk of VTE than patients not taking lipid lowering drugs [150,151,152], although with controversial results [153]. The mechanisms by which statins reduce VTE risk are not yet understood, although their capability of modifying endothelial function lowering the inflammatory response may provide a rational link with HDL.

Finally, a recent study investigating real-world features associated with cancer-related VTE showed that serum creatinine can be considered an independent indicator of increased VTE risk during platinum-based chemotherapy [154], confirming previous findings demonstrating that estimated glomerular filtration rate is associated with an increased VTE risk in cancer outpatients treated with platinum compounds, even under normal serum creatinine values [155].

From all the above, it is clear that the growing big health-data scenario may provide powerful tools to mine knowledge from EHRs, to identify novel predictive biomarkers whose combination could be used for VTE risk assessment and targeted prophylaxis.

## 3. Current Models for VTE Risk Prediction in Ambulatory Cancer Patients

Cancer-associated risk factors described so far, together with routinely collected demographic, clinical, and biochemical data, have all been used to design clinical risk models (Table 1). Notwithstanding, the issue of VTE risk prediction in chemotherapy-treated cancer outpatients is still far from being resolved.

At present, the most used RAM designed to stratify cancer outpatients before the start of chemotherapy, is the Khorana score, a simple and user friendly tool that combines routinely available parameters to assign patients to different classes of VTE risk [22] (Table 1). Based on preliminary results, the use of the Khorana score at a cutoff ≥3 was initially proposed in a thromboprophylaxis guidance statement [156]. However, later studies disclosed its low sensitivity for certain tumor types, like lung [23,24,61,157,158] or pancreatic [159] cancer. Moreover, the high proportion of patients (>50%) falling into the intermediate risk category represented a serious drawback. In fact, while the decision to treat low-risk or high-risk patients is fairly easy to be taken, how to handle patients in the intermediate-risk category represents a big challenge for physicians. Thus, recent randomized trials have adopted the use of a cutoff ≥2 to stratify cancer patient candidates for thromboprophylaxis [160,161]. This is the case of the CASSINI study (ClinicalTrials.gov Identifier: NCT02555878), whose interim results demonstrated that rivaroxaban significantly reduced VTE and VTE-related death during the on-treatment period of at-risk ambulatory cancer patients selected on the basis of a Khorana score ≥2 [160]. The same selection criterion was used in the AVERT study (Apixaban for the Prevention of Venous Thromboembolism in Cancer Patients; ClinicalTrials.gov Identifier: NCT02048865), whose results suggest that apixaban may significantly lower VTE incidence in intermediate-to-high-risk ambulatory cancer patients starting chemotherapy, although at a higher rate of major bleeding compared to placebo [161]. The feasibility of a revised cutoff at ≥2 points was recently confirmed in a meta-analysis specifically designed to estimate the performance of the Khorana score [162]. Using a threshold of 2 points rather than the conventional 3 points, in fact, it was observed a substantial increase of the proportion of high-risk patients (from 17% to 47%), paralleled by a reduction of the absolute VTE risk (from 11% to 9%). In the real-world clinical practice, however, the Khorana risk score was shown to have no influence on the therapeutic decision to start prophylaxis in the CAT AXIS, a multicentered cross-sectional case vignette study on clinical practice in France [163].

To improve its predictive performance, the original Khorana score was modified by adding either chemotherapy agents, such as platinum-based regimens and gemcitabine, as in the case of the PROTECT score, that resulted in an improved ability to identify patients at higher risk for VTE [27], or biomarkers [25] (Table 1). This last scoring system developed by the Vienna CATS investigators [25], introduced the evaluation of both D-dimer (with a cut-off of 1.44 μg/mL) and sP-sel (with a cut-off of 53.1 ng/mL), which appeared to considerably improve the risk prediction of VTE [25]. A prospective cohort study provided a direct comparison of the performance of the four clinical and biomarker-based prediction scores for VTE in patients with advanced solid cancer receiving chemotherapy [164]. The authors found a poor overall discriminatory performance of all the scores, and attributed such a result to the findings of the multivariable analysis. However, the Vienna CATS and the PROTECHT scores performed better than the other two scores, probably because the predictive performance of the Vienna CATS score appeared to be mainly driven by the predictive performance of D-dimer levels and that of the PROTECHT score by the type of chemotherapy. More recently, a risk assessment tool within the COMPASS–CAT study (Prospective Comparison of Methods for thromboembolic risk assessment with clinical Perceptions and AwareneSS in real life patients-Cancer Associated Thrombosis) which included in the score patient’s co-morbidities, cancer-related and treatment-related factors, was applied to outpatients with selected cancer types, such as breast, colon, lung, or ovarian cancer after antineoplastic treatment initiation [165]. This RAM showed that after initiation of anticancer treatment, patient-related risk factors were the major determinants for the risk of cancer-associated VTE and that co-morbidities were associated with a five-fold increase of VTE risk, which increased even more when co-morbidities and cardiovascular risk factors were summed together [165]. A similar approach was pursued in the ONKOTEV study, which aimed at analyzing a mixed population of cancer patients typically treated in an outpatient setting. In this prospective observational study a Khorana score >2, personal history of VTE, metastatic disease, and vascular/lymphatic macroscopic compression independently showed a significant association with VTE and were, thus, used to set up a multi-items score (assigning one point to each variable), which significantly improved the original Khorana score [166]. Conversely, Muñoz Martín and colleagues recently proposed a new index, the TiC-Onco risk score, based on patients’ clinical and genetic risk factors for thrombosis [167]. Although interesting and definitely an improvement of the original Khorana score, the index cannot be performed at any laboratory and sensibly increases the overall costs for VTE risk assessment, thus reducing the clinical applicability of the model.

In the attempts to simplify risk assessment for VTE in patients with cancer, Pabinger and co-workers developed a simple model that eliminated many of the variables included in the Vienna CATS score, and focused only on tumor-site and D-dimer concentration (as a continuous variable) [168]. This novel clinical prediction model was able to discriminate between patients who did and did not develop VTE during a 6-month follow-up [168]. The application of this tool to a dataset of cancer patients enrolled in the Institutional Biobanks of our research group resulted in similar c statistic and analytical performance to those reported by Pabinger [168], even with a different D-dimer assay, indicating large clinical applicability of the proposed nomogram [169].

## 4. Artificial Intelligence for Cancer-Associated Thrombosis Risk Assessment

In recent years, the approach to medicine has substantially changed under the pressure of a growing availability of EHRs and the demand to provide precision medicine. Oncology is one of the fields mostly demanding for precision medicine in a “big data” world, as highlighted in the 2016 report of the Blue Ribbon Panel of the Cancer Moonshot initiative that recommended to mine past patient data for predicting future patient outcomes and for minimizing cancer treatment’s debilitating side effects [170]. However, the general problem of precision medicine is represented by the huge amount of clinical variables to consider in order to extract knowledge from the growing volumes of digital data and highlights the urgent need for a new generation of computational theories and tools [171].

In this context, we recently hypothesized that AI would be a solid instrument to build a predictive tool for VTE risk assessment in chemotherapy-treated cancer outpatients. Thus, we applied a combined approach of kernel machine learning (KML) and random optimization (RO) techniques to design and validate a set of VTE predictors capable of exploiting significant patterns in routinely collected demographic, clinical and biochemical data [172].

To test our hypothesis, age, sex, tumor site and stage, hematological attributes (including blood cell counts, hemoglobin, neutrophil- and platelet-lymphocyte ratios), fasting blood lipids, glycemic indexes, liver and kidney function, BMI, Eastern Cooperative Oncology Group (ECOG) performance status, supportive and anti-cancer drugs, were all analyzed in a cohort of 1433 cancer out-patients at the start of a new chemotherapy regimen. Variables were clustered into groups according to clinical significance. The algorithm was devised using a training set, and a testing set was used to compute the final performance of our risk predictors [172]. Moreover, a validation set was used to internally validate the approach used [173].

To find the best combination of clinical attributes, the performance of predictors was maximized using RO by a 3-fold cross validation technique on the training set. RO is a method that can be used also on functions that are not continuous or differentiable. Some interesting consideration could be drawn from this novel methodological approach. First, the analysis of clinical/biochemical variables identified several risk factors that were not previously considered in VTE risk models (i.e., blood lipids or glucose). Second, models employing additional clinical attributes showed better measures and positive likelihood ratios than the Khorana score [171], and this was verified both on the training and on the validation set. This technique, which optimizes the relative importance of groups of clinical attributes, appears extremely useful in selecting VTE risk predictors [172], and represents an inexpensive approach that can be easily adapted to different local situations/populations.

**Figure 3:**
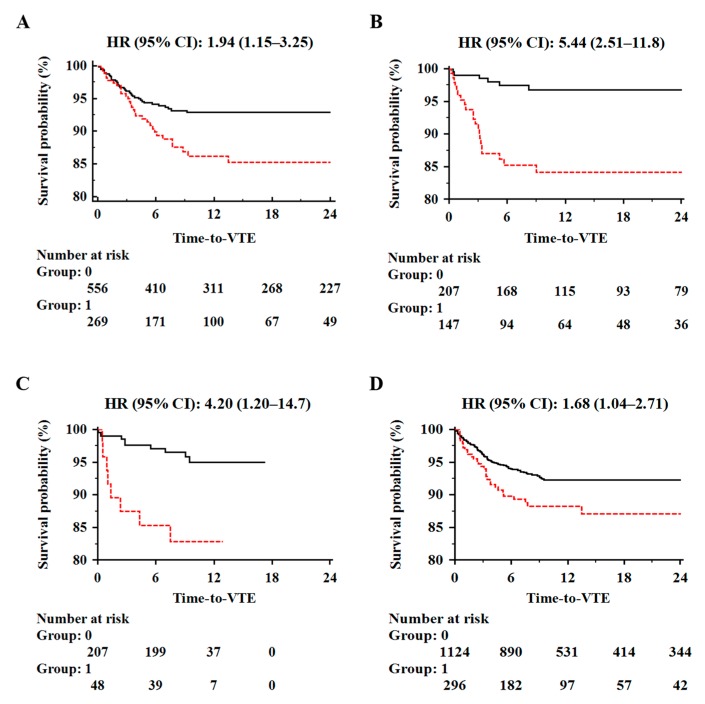
Kaplan–Meier curves of VTE-free survival time of chemotherapy-treated ambulatory cancer patients in the training (Panel **A**, *n* = 825), testing (Panel **B**, *n* = 354) and validation set (Panel **C**, *n* = 255). Comparison between patients with low (Group: 0; solid black line) or high (Group: 1; dotted red line) risk of VTE based on the combined model predictor. Patients stratification based on the Khorana score ≥2 is also given for comparison (Panel **D**).

As the performance of VTE predictors could be further enhanced, we also designed a combined model incorporating the two best predictors of ten independent runs, which resulted in a significant improvement of VTE risk prediction performance over the Khorana score, even when the latter was used at a cutoff ≥2 (Figure 3) [169,173].

The possibility to incorporate the devised algorithms into an online risk calculator—with a graphical interface supporting the oncologists in the critical phase of VTE risk assessment—and the fact that all the variables are usually included in the workout routine of cancer patients (and can be easily extracted from EHRs) confer further strength to this approach.

## 5. Conclusions and Future Perspectives

Automated predictive models for VTE risk prediction and stratification represent innovative clinical decision support systems that are experiencing a significant boost thanks to the rapid progress of ICT (Information and Communication Technologies) tools allowing the development of customized interfaces extracting data from EHRs. Customized and evidence-based management of patients on the basis of computerized systems, could provide a real-time VTE risk calculation guiding clinicians in the decision making process [174]. Besides, in the application of predictive analysis techniques in health sectors, the use of Big Data sources represents a relevant factor in terms of effectiveness and cost-efficacy towards a personalized medicine-based approach [175,176]. The creation of a platform for mining knowledge and of learning health systems capable of delivering informative clinical evidence, will ensure predictive models of quality are obtained [176]. In this context, we believe that an optimal VTE risk prediction might be achieved by means of a combination of AI/machine learning and EHRs big-data, which offer a revolutionary way of practicing evidence-based medicine in a context of precision medicine as advocated by the Cancer Moonshot initiative.

## Figures and Tables

**Table 1:** Comparison of the characteristics of risk assessment models (RAM) for cancer-associated venous thromboembolism (VTE).

RAM	Score Items	n. of Patients	Type of VTE	c-Statistic	HR	Reference
Khorana score (KS)	Site of cancer, platelet count, leukocyte count, hemoglobin level or use of red cell growth factors, BMI ≥35	Derivation cohort, *n* = 2701	Symptomatic	0.7 for both cohorts	NA	[22]
		Validation cohort, *n* = 365				
Vienna CATS score	Adds soluble P-selectin and D-dimer to KS	*n* = 819	Symptomatic	NA	1.9 per 1 point increase	[25]
PROTECHT score	Adds cisplatin/carboplatin-based chemotherapy or gemcitabine to KS	Placebo arm, *n* = 381	Unclear	NA	NA	[27]
		Nadroparin arm, *n* = 769				
ONKOTEV score	Khorana score >2, personal history of VTE, metastatic disease, vascular/lymphatic macroscopic compression	*n* = 843	Symptomatic/incidental	0.719 at 3 months 0.754 at 6 months	Score = 1: 3.29 Score = 2: 6.54 Score > 2: 13.74	[166]
COMPASS-CAT score	Anthracycline or anti-hormonal therapy, time since cancer diagnosis, central venous catheter, stage of cancer, presence of cardiovascular risk factors, recent hospitalization for acute medical illness, personal history of VTE and platelet count.	*n* = 1023	Symptomatic	0.850	NA	[165]
Tic-ONCO score	Adds genetic risk score to KS	*n* = 391	Symptomatic	0.73	+LR = 1.69	[167]
CATS nomogram	Site of cancer and D-dimer	CATS cohort, *n* = 1423	Symptomatic/incidental	0.66 in CATS	NA	[168]
		MICA cohort, *n* = 832		0.68 in MICA		

HR: Hazard Ratio; +LR: Positive Likelihood Ratio; CATS: Vienna Cancer and Thrombosis Study; MICA: Multinational Cohort Study to Identify Cancer Patients at High Risk of Venous Thromboembolism; NA: Not Applicable.
